# Practical Protocols for Solid-Phase Peptide Synthesis 4.0

**DOI:** 10.3390/mps5060085

**Published:** 2022-10-24

**Authors:** Beatriz G. de la Torre, Fernando Albericio

**Affiliations:** 1 KRISP, College of Health Sciences, University of KwaZulu-Natal, Durban 4001, South Africa; 2 School of Chemistry and Physics, University of KwaZulu-Natal, Durban 4001, South Africa; 3 CIBER-BBN, Networking Centre on Bioengineering, Biomaterials and Nanomedicine, Department of Organic Chemistry, University of Barcelona, 08028 Barcelona, Spain

According to the Food and Drug Administration (FDA), there are two kinds of drugs, namely New Chemical Entities (NCEs) and Biologics. The former are well-characterized molecules or ions responsible for the physiological or pharmacological action of the drug substance [1]. NCEs are associated with the simple chemical concept of “pure substance”. In contrast, Biologics are generally large, complex molecules. They are produced by biotechnology and are often more difficult to characterize than NCEs [2].

Peptides and Oligonucleotides (TIDES), although very often large and complex molecules, are chemically produced and belong to the NCE class. Due to their inherent complexity, only a few decades ago the idea of having a peptide such as the recently approved tirzepatide (Mounjaro^TM^) with a backbone of 39 amino acids and a pending branch formed by four moieties produced at a multi Kg scale was unimaginable [3]. Similarly, inclisiran (Leqvio^TM^), which was approved in 2021, is a double-stranded siRNA—with 21 and 23 ribonucleosides for the sense and antisense strands, respectively. In addition, the sense strand is linked to a short dendrimer bearing N-acetylgalactosamine (GalNAc) [4].

The entry of large and complex TIDES-based drugs into the market has been possible thanks to a simple discovery, namely solid-phase synthesis. During these days, we are celebrating the 60th anniversary of the first public announcement of the Solid-Phase Peptide Synthesis (SPPS) technique at the Federation Meeting held in Atlantic City in 1962 by the Nobel Laureate R. Bruce Merrifield. The first publication appeared a year after this event [5]. Just two years later, Letsinger published the application of Merrifield’s methodology to oligonucleotide synthesis [6] (SPPS for peptides and SPOS for oligonucleotides), and so began the era of the chemical synthesis of complex biomolecules using the solid-phase approach. Without this technique, the current panorama of TIDES in the pharmaceutical industry would be very different. 

Merrifield’s idea was very simple: growing the peptide sequence from the C- to the N-terminal using a functionalized insoluble polymer (resin, solid support) as a permanent protecting group of the C-carboxylic acid. Thus, the rest of the amino acids are sequentially incorporated bearing a temporal protecting group for the α-amino function, which is removed after each step, and a permanent protecting group for the side chains if necessary. These protecting groups are removed at the end of the stepwise elongation at the same time as the peptide is released from the solid support. The use of the solid protecting group allows the use of an excess of reagents, thereby facilitating excellent yields [7].

The strategy had several critics and skeptics in the beginning, mainly established peptide chemists [8]. However, with time, SPPS has become the method of choice for the preparation of peptides on an mg scale in a research mode and on a multiKg scale as part of Active Pharmaceutical Ingredients [9,10]. 

According to the excellent database PepTherDia, promoted by D’Aloisio et al., currently, there are 113 peptides on the market [11,12]. 

Table 1, Table 2 and Table 3 shows approvals by the FDA between 1 January 2016, and 30 September 2022, of peptide-based therapeutic/diagnostic drugs (20), radioactive peptide-based thera-/dia-gnostic agents (6), and antibody-drug conjugates (ADCs), where the drug (cytotoxic) is a peptide (4), respectively. In total, 30 therapeutic/diagnostic peptide-based drugs have been approved over these years. At this point, it is important to highlight that the “definition” of peptide associated with Table 1 and Table 2 is somewhat wider than that used in PepTherDia. The peptides shown in Table 1 and Table 2 are formed by at least two amino acids, which are bound, preferably by a peptide bond.

This scientific “miracle” started in the Merrifield laboratory at Rockefeller University. However, keeping the same original concept, it has been possible to optimize both the synthesis and purification steps, allowing the preparation of peptide-based APIs on a multiKg scale to feed the pharmaceutical industry. This achievement has been possible thanks to the contribution of a very large number of colleagues, working both in academia and industry. Many of these chemical advances have been published in scientific journals, but they are scattered throughout the vast network of scientific information. 

This Special Issue of Materials and Protocols, entitled “Practical Protocols for Solid-Phase Peptide Synthesis 4.0”, seeks to bring together the state of the art in SPPS in a single publication. 

“Practical Protocols for Solid-Phase Peptide Synthesis 4.0” will cover all the practical aspects of peptide synthesis, purification, and characterization: resins, protecting groups, coupling agents, cleavage cocktails and their scavengers, solvents for synthesis and purification, synthesis of linear, cyclic, stapled, branched peptides, with post-translational modifications, peptide-drug conjugates, and all purification and analysis methods. This issue will pay particular attention to the great challenge faced by chemists, which is no other than maintaining the quality of synthesized products while making processes greener, thus contributing to a more sustainable world.

Hopefully, the peptide scientific community will be able to share in this Special Issue with the rest of those little tricks that facilitate the synthesis/production of the target peptide in excellent yields and purity, and that this knowledge benefits a wider community.

## Figures and Tables

**Table 1:** Peptide-based drug/diagnostic approved by the FDA between 1 January 2016 and 30 September 2022 [13,14,15,16,17,18,19].

Active Ingredient ^a^	Trade Name ^a^	Indication	Year of Approval
Lixisenatide	Adlyxin^TM^	Type 2 diabetes	2016
Abaloparatide	Tymlos^TM^	Osteoporosis	2017
Angiotensin II	Giapreza^TM^	Control of blood pressure	2017
Etelcalcetide	Parsabiv^TM^	Hyperparathyroidism	2017
Macimorelin	Macrilen^TM^	Adult growth hormone deficiency	2017
Plecanatide	Trulance^TM^	Chronic idiopathic constipation	2017
Semaglutide	Ozempic^TM^	Type 2 diabetes	2017
Afamelanotide	Scenesse^TM^	To prevent skin damage and pain after exposure to the sun.	2019
Bremelanotide	Vyleesi^TM^	Hypoactive sexual desire in premenopausal women	2019
Setmelanotide	Imcivree^TM^	Obesity and hunger	2020
Dasiglucagon	Zegalogue^TM^	Hypoglycemia in diabetes	2021
Difelikefalin	Korsuva^TM^	Pruritus	2021
Melphalan flufenamide	Pepaxto^TM^	Multiple myeloma	2021
Odevixibat	Bylvay^TM^	Pruritus	2021
Pegcetacoplan	Empaveli^TM^	Paroxysmal nocturnal hemoglobinuria	2021
Voclosporin	Lupkynis^TM^	Lupus nephritis	2021
Vosoritide	Voxzogo^TM^	Achondroplasia (Dwarfism)	2021
Gadopiclenol	Elucirem™	Diagnostic of lesions in the central nervous system	2022
Terlipressin	Terlivaz^TM^	Low blood pressure	2022
Tirzepatide	Mounjaro^TM^	Type 2 diabetes and obesity	2022

^a^ Trade name used in the USA.

**Table 2:** Radioactive peptide-based thera-/dia-gnostic agents approved by the FDA between 1 January 2016 and 30 September 2022 [13,14,15,16,17,18,19].

Active Ingredient ^a^	Trade Name ^a^	Indication	Year of Approval
Lutetium 177 DOTA-TATE	Lutathera^TM^	Theragnostic for neuroendocrine tumors	2018
Gallium 68 DOTA-TOC		Diagnostic for tumors	2019
Copper 64 dotatate	Detectnet^TM^	Diagnostic for neuroendocrine tumors	2020
Gallium 68 PSMA-1		Diagnostic for prostate cancer	2020
Piflufolastat F-18	Pylarify^TM^	Diagnostic for prostate cancer	2021
Lutetium 177 vipivotide tetraxetan	Pluvicto^TM^	Theragnostic for prostate cancer	2022

^a^ Trade name used in the USA.

**Table 3:** ADCs approved by the FDA between 1 January 2016 and 30 September 2022 in which the drug is a peptide [13,14,15,16,17,18,19].

Active Ingredient ^a^	Trade Name ^a^	Indication	Year of Approval
Enfortumab vedotin-ejfv ^b^	Padcev^TM^	Cancers expressing Nectin-4	2019
Polatuzumab vedotin-piiq ^b^	Polivy^TM^	Diffuse large B-cell lymphoma	2019
Belantamab mafodotin-blm	Blenrep^TM^	Multiple myeloma	2020
Tisotumab vedotin-tftv ^b^	Tivdak ^TM^	Cervical cancer	2021

^a^ Trade name used in the USA; ^b^ The linker contains a peptide.
